# The Zelnorm epidemiologic study (ZEST): a cohort study evaluating incidence of abdominal and pelvic surgery related to tegaserod treatment

**DOI:** 10.1186/1471-230X-12-171

**Published:** 2012-11-30

**Authors:** John D Seeger, Sherry Quinn, David L Earnest, Anthony Lembo, Braden Kuo, Elena Rivero, Alexander M Walker

**Affiliations:** 1 Brigham & Women’s Hospital and Harvard Medical School, Boston, MA, USA; 2 Department of Epidemiology, Harvard School of Public Health, Boston, MA, USA; 3 OptumInsight, Waltham, MA, USA; 4 Novartis Pharmaceuticals Corporation, East Hanover, NJ, USA; 5 Beth Israel Medical Center, Boston, MA, USA; 6 Gastrointestinal Unit, Massachusetts General Hospital, Boston, MA, USA; 7 Novartis Farmaceutica, Barcelona, Spain; 8 World Health Information Science Consultants (WHISCON), Newton, MA, USA; 9 1620 Tremont St, Suite 3300, Boston, MA, 02120, USA

**Keywords:** Abdominal surgery, Tegaserod, Cohort study

## Abstract

**Background:**

Pre-marketing clinical studies of tegaserod suggested an increased risk of abdominal surgery, particularly cholecystectomy. We sought to quantify the association between tegaserod use and the occurrence of abdominal or pelvic surgery, including cholecystectomy.

**Methods:**

This cohort study was conducted within an insured population. Tegaserod initiators and similar persons who did not initiate tegaserod were followed for up to six months for the occurrence of abdominal or pelvic surgery. Surgical procedures were identified from health insurance claims validated by review of medical records. The incidence of confirmed outcomes was compared using both as-matched and as-treated analyses.

**Results:**

Among 2,762 tegaserod initiators, there were 94 abdominal or pelvic surgeries (36 gallbladder): among 2,762 comparators there were 134 abdominal or pelvic surgeries (37 gallbladder) (hazard ratio HR] = 0.70, 95% confidence interval [C.I.] = 0.54-0.91 overall, HR = 0.98, 95% C.I. = 0.62-1.55 for gallbladder). Current tegaserod exposure compared to nonexposure was associated with a rate ratio [RR] of 0.68 (95% C.I. = 0.48-0.95) overall, while the RR was 0.99 (95% C.I. = 0.56-1.77) for gallbladder surgery.

**Conclusions:**

In this study, tegaserod use was not found to increase the risk of abdominal or pelvic surgery nor the specific subset of gallbladder surgery.

## Background

Tegaserod (Zelnorm®/Zelmac®) is a selective, partial serotonin type 4 (5HT_4_) receptor agonist that has therapeutic benefit in patients with irritable bowel syndrome with constipation (IBS-C) and chronic idiopathic constipation [1-4]. It was approved for clinical use in the U.S. and 61 other countries. A review of the pre-marketing studies of tegaserod for treatment of IBS-C indicated an imbalance in the number of abdominal surgeries observed among the tegaserod treated patients (9/2,965; 0.3%) relative to patients receiving placebo (3/1,740; 0.2%), and an imbalance in cholecystectomy among tegaserod recipients (5 of 2,965; 0.17%) relative to placebo (1 of 1,740; 0.06%) [5]. Although abdominal surgery and cholecystectomy occurred, respectively, 1.7 and 3 times as often in the tegaserod-treated group, the rarity of these procedures during the clinical trials meant that the apparent difference between treatment groups could reasonably be attributed to chance. Furthermore, clinical review of the medical records for these cases suggested alternative etiologies for some of the procedures suggesting that the difference in incidence between tegaserod and placebo groups was even smaller than the total numbers indicated [6].

Our aim was to quantify the association between tegaserod exposure and the occurrence of abdominal or pelvic surgery including gallbladder surgery. We conducted the Zelnorm Epidemiologic Study (ZEST) as a post-marketing study within a health insurance database that reflected use of tegaserod as part of usual clinical practice for patients across the United States [7].

Since this study was completed, results of pooled safety analyses of 29 placebo-controlled clinical studies (11,614 patients treated with tegaserod and 7,031 treated with placebo), revealed a small yet statistically significant (13 tegaserod vs. 1 placebo, p = 0.024) imbalance in the number of patients having a cardiovascular ischemic event (myocardial infarction, stroke, unstable angina, etc.). Following a review of these data with health authorities, the marketing and sales of Zelnorm®/Zelmac® were suspended in the U.S.A, and at least twenty other countries. Subsequent to this action, the epidemiological data from patients taking tegaserod and comparator groups were evaluated for evidence of adverse cardiovascular ischemic events, and no increased risk was found for tegaserod [8,9]. Tegaserod does remain available in the US and four other countries under a special compassionate use program and is also still marketed in 5 other countries. In addition, other drugs with 5HT_4_ agonist activity are under clinical trial evaluation or submitted for regulatory approval. Accordingly, the results of this study remain pertinent to patient care.

## Methods

This cohort study was conducted within the Ingenix Research Database, which includes persons with both commercial (employed people and their dependents) and Medicare supplement health insurance and is geographically dispersed across the US. The database includes information on enrollee age, sex, enrolled dates and claims for reimbursement for all types of health care services. Each reimbursement claim is linked to an encrypted enrollee identifier and contains patient diagnoses, coded according to the International Classification of Diseases, Ninth Revision (ICD-9), with medical procedures coded using one of the following: ICD-9 procedure codes, Current Procedural Terminology codes, or Health Care Financing Agency Common Procedure Coding System level II codes. Pharmacy claims for dispensing of prescription drugs are also included in the database, with the drug name in addition to formulation and number of days supplied. The data are audited, and the validity of the claims has been previously documented [10].

The cohorts consisted of tegaserod initiators and comparators who were contemporary non-initiators of tegaserod. Patients were counted as tegaserod initiators if a first known dispensing was preceded by at least six months in the database, and comparators similarly needed at least six months of preceding database enrollment for eligibility. The comparators were chosen to have similar characteristics to the tegaserod initiators with respect to numerous baseline characteristics and claims for medical services beyond calendar time. This study was conducted across the first year of tegaserod availability in the US, and included initiators in each calendar quarter (4th quarter 2002, 1st quarter 2003, 2nd quarter 2003, or 3rd quarter 2003) who were selected, along with a pool of patients who did not initiate tegaserod within the same calendar quarter.

We identified tegaserod initiators and a pool of potential comparators who were selected on the basis of a diagnosis code for functional digestive disease (ICD-9: 564.x), or other abdomen or pelvis symptoms (ICD-9: 789.x), or gastrointestinal system symptoms (ICD-9: 787.x) contemporaneous with the tegaserod initiators. We characterized patients with respect to a wide range of medical and pharmacy insurance claims (including demographics, healthcare utilization, calendar time, gastrointestinal care correlates, surgical procedures, and empirically-identified variables) during the 6-months preceding the initial tegaserod dispensing (or, for the non-initiators, a date that was assigned at random from among the tegaserod initiation dates), and a multivariable balancing technique (propensity score matching) was used to produce similar groups [11,12]. The cohorts were formed on a quarterly basis and pooled for the outcome analysis [13]. The propensity score modeling and the matching based on it was conducted separately each calendar quarter, so that each quarter’s model could vary both with respect to the variables included and the weight of each variable, accounting for changes in the way tegaserod was prescribed over the time of the study. A comparator patient was selected for each of the tegaserod initiators on the basis of a narrow and fixed caliper of propensity score (0.01).

Tegaserod initiators and comparators were followed for up to 183 days from the date of cohort entry (less if members disenrolled prior to the end of the 183 days) for the occurrence of abdominal surgery. We identified potential surgeries through insurance claims and sought medical records relating to the surgeries identified in order to confirm that the procedure met the study definition of surgery. Blinded medical records were all reviewed by an independent clinical expert panel composed of four physicians with a range of clinical expertise who arrived at a consensus about whether each potential case met the study definition of abdominal or pelvic surgery, the type of surgery and its date. (Appendix A) Study outcomes included in this report and analyses involving them are based only on outcomes that were confirmed through this process.

Counts of abdominal or pelvic surgery and gallbladder surgery among tegaserod initiators and comparators were summarized along with measures of comparison between the cohorts and associated confidence intervals. The comparisons incorporate covariate adjustment for baseline demographics, health conditions and health service utilization, and provide both “as-matched” analyses that correspond to intent-to-treat analyses, and “as-treated” analyses that update tegaserod exposure according to pharmacy claims that indicated dispensing of tegaserod during follow-up.

The incidence of abdominal or pelvic surgeries during the follow-up of the tegaserod initiator or comparator cohorts was analyzed using proportional hazards regression, with separate models developed for abdominal and pelvic surgeries and for gallbladder surgeries. We also incorporated covariate adjustment (even though the propensity score matching process largely removed differences between the groups). This adjustment served both to address the small remaining differences between tegaserod users and comparators with respect to these variables, and to show the association between these variables and the risk of abdominal and pelvic surgeries or gallbladder surgeries. Covariates used for this adjustment were selected in a stepwise process in separate models to evaluate prediction of abdominal or pelvic surgery or gallbladder surgery.

To account for this change in exposure during follow-up, we conducted an analysis that combined person-time into similar categories of tegaserod exposure (current, recent, past, or non-user). The “current tegaserod” category started the day after a dispensing of tegaserod and continued through the end of the number of days supplied by that dispensing plus 14 days. The “recent tegaserod” category started at the end of the “current tegaserod” category and continued for up to 28 days, and the “past tegaserod use” category characterizes all of the time thereafter. The “non-user” category was for persons who had no prior or current use of tegaserod within the study time frame. Each new dispensing of tegaserod reset the use category to “current tegaserod” and the other categories followed. All surgeries representing study outcomes that occurred during the follow-up period were assigned to one of the four tegaserod-use categories depending on the exposure status of the person at the time of the event.

A Poisson regression model was used to compare the incidence rate of surgery across tegaserod exposure categories incorporating covariate adjustment, since changes in therapy may be related to patient characteristics, so that exposure categories may not be as well balanced with respect to covariates as were the originally-matched cohorts. Covariates for this analysis arose from those selected for adjustment in the “as balanced” analysis.

Analyses were conducted using SAS (version 8). The study protocol was approved by the New England Institutional Review Board.

## Results and discussion

There were 2,853 eligible tegaserod initiators across the four study accrual periods, of which 2,762 (96.8%) were matched to 2,762 comparators. The variables included in the propensity score varied according to the matching block, and included an average of 77 (range 52–107) variables. The resulting score was highly discriminating between tegaserod initiators and non-initiators (average c-statistic = 0.90). Characteristics of the tegaserod initiators and their matched comparators did not differ substantially for any of the characteristics that were part of the propensity score (demographics, pre-existing diagnoses or medical conditions, procedures, prior drug use, and baseline healthcare utilization). (Table 1) We did not exclude people with claims indicating abdominal surgery in the six-month baseline period (preceding cohort entry), and the occurrence of claims for abdominal surgery during baseline did not differ substantially between the cohorts (17.2% vs 15.2% for any abdominal surgery and 1.7% vs 1.5% for gallbladder surgery).

**Table 1:** **Characteristics of tegaserod initiators and comparators at baseline**^**1**^

**Characteristic or Condition**	**Tegaserod initiators (N = 2,762)**		**Comparison cohort (N = 2,762)**	
	**N**	**%**	**N**	**%**
**Age group**				
0-29	386	14.0%	386	14.0%
30-49	1,416	51.3%	1,412	51.1%
50+	960	34.8%	964	34.9%
**Sex**				
Female	2,496	90.4%	2,435	88.2%
**Region**				
Northeast	73	2.6%	70	2.5%
South & Southeast	1,256	45.5%	1,312	47.5%
Midwest	1,228	44.5%	1,159	42.0%
West	205	7.4%	221	8.0%
**Select Baseline Diagnoses**				
Abdomen / Pelvis Symptoms (ICD-9 789)	1,413	51.2%	1,417	51.3%
Irritable Bowel Syndrome (ICD-9 564.1)	1,150	41.6%	1,117	40.4%
Constipation (ICD-9 564.0)	1,027	37.2%	967	35.0%
GI System Symptoms (ICD-9 787)	886	32.1%	1,050	38.0%
**Select Baseline Procedures**				
Surgical Pathology (CPT 88305)	696	25.2%	700	25.3%
Colon Procedures (CPT	643	23.3%	665	24.1%
CT Scan Abdomen (CPT 74150–74175)	413	15.0%	426	15.4%
Abdominal Ultrasound (CPT	381	13.8%	401	14.5%
Gallbladder Radiography (CPT	84	3.0%	66	2.4%
Gallbladder Surgery (CPT	46	1.7%	42	1.5%
**Baseline Drug Classes Dispensed**				
Proton Pump Inhibitors	1,029	37.3%	1,059	38.3%
Analgesics, Narcotics	955	34.6%	998	36.1%
Serotonin Specific Reuptake Inhibitor	692	25.1%	686	24.8%
Laxative Drugs	533	19.3%	522	18.9%
**Baseline Healthcare Utilization**	Mean	Median	Mean	Median
Total Cost ($)	4,319.40	2,127.77	4,750.59	2,254.55
Physician Costs (US$)	1,501.48	828.12	1,576.03	849.34
Cost of Pharmaceuticals (US$)	923.33	461.04	937.55	405.71
Gastroenterologist Visits	1.6	1	1.6	0
Number of Drug Classes Dispensed	7.9	7	7.8	7

^1^ Defined as the six-month period preceding start of follow-up.

We identified 456 potential abdominal or pelvic surgeries on the basis of claims data during the 6-month follow-up of the matched cohorts and sought the medical records for 448 (98.2%) of them, excluding only those that could be ruled out on the basis of a claims review alone. We obtained 376 of the 448 (83.9%) requested medical records, and the remainder could not be obtained. The expert clinical review panel confirmed that 228 of the 376 (60.6%) potential abdominal or pelvic surgeries identified met the study outcome definition (for the subset of gallbladder surgeries, 73 of the 92 (79.3%) met the study outcome definition). The surgeries not meeting the study definition tended to be ones performed under local anesthesia or non-surgical abdominal or pelvic procedures. Only confirmed surgeries meeting study definition were included in outcome analyses.

**Figure 1:**
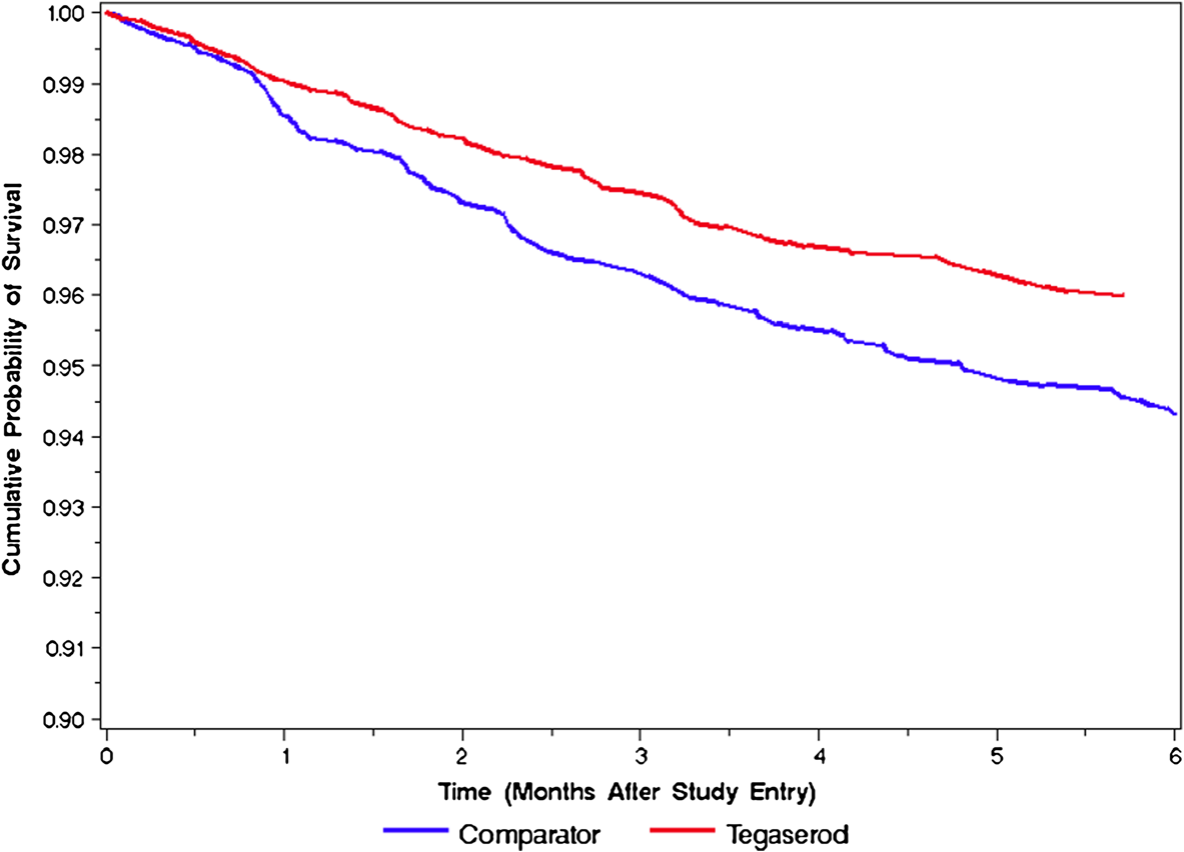
**Time to First Abdominal or Pelvic Surgery(As**-**Matched Analysis)**.

**Figure 2:**
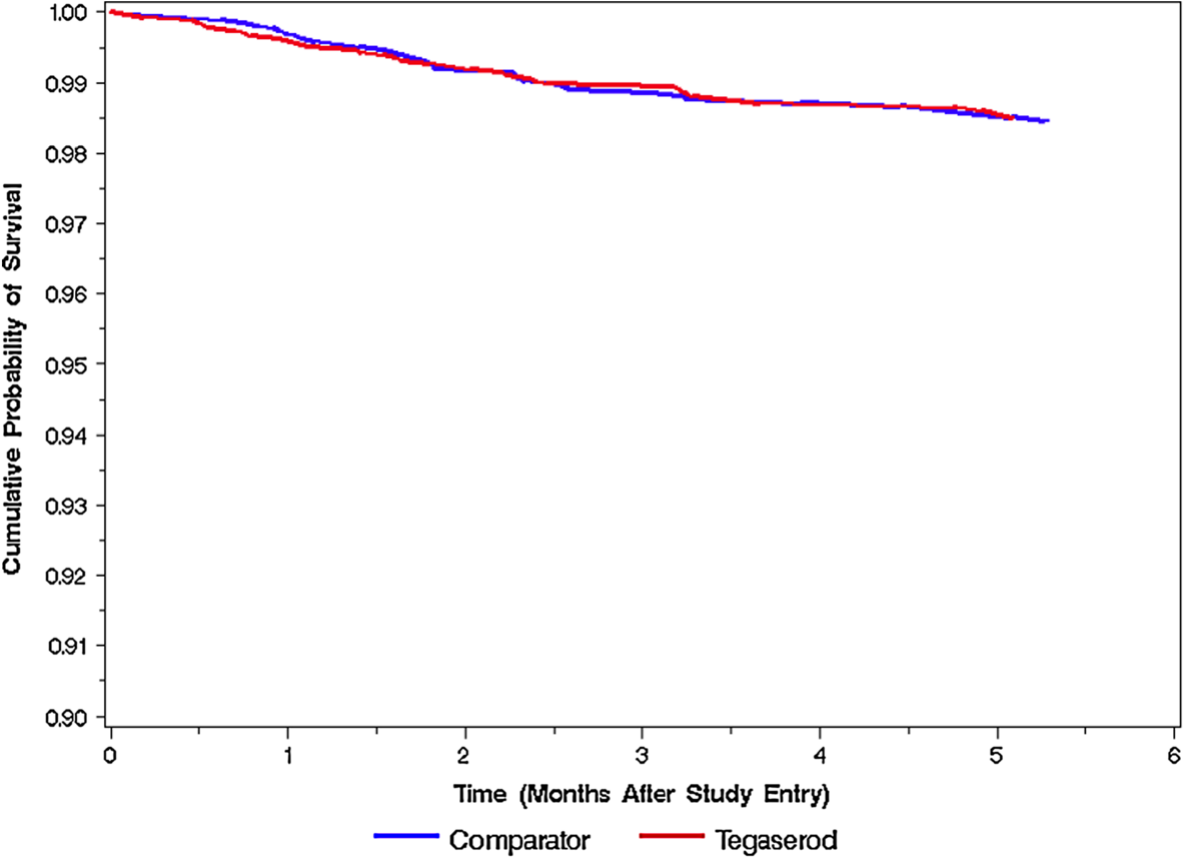
**Time to First Gallbladder Surgery(As**-**Matched Analysis).**

The “as matched” analysis was conducted separately for all abdominal surgeries combined (Table 2 and Figure 1) and for gallbladder surgeries (Table 3 and Figure 2). The incidence of all surgeries combined was lower in the tegaserod initiating cohort than it was in the comparator cohort (HR = 0.70, 95% C.I. = 0.54-0.91). Adjusting for baseline risk factors did not substantially alter this association (adjusted HR = 0.71, 95% C.I. = 0.55-0.93). The incidence of gallbladder surgeries was almost identical in the two cohorts (HR = 0.98, 95% C.I. = 0.62-1.55), and this was also unaffected by adjustment for baseline risk factors (adjusted HR = 0.99, 95% C.I. = 0.62-1.58).

**Table 2:** Incidence of abdominal or pelvic surgeries among tegaserod initiators and comparators (as-matched analysis)

**Variable**	**Events**	**Person years**	**I**.**R**.^**1**^	**95% C**.**I**.		**Hazard ratio**^**2**^	**95% C**.**I**.		**Hazard ratio**^**3**^	**95% C**.**I**.	
				**Low**	**High**		**Low**	**High**		**Low**	**High**
**Exposure**											
Tegaserod Initiators	94	1,101	0.09	0.07	0.10	0.70	0.54	0.91	0.71	0.55	0.93
Comparators	134	1,100	0.12	0.10	0.14	1.00			1.00		
**Gender**											
Female	207	1,963	0.11	0.09	0.12	1.00			1.00		
Male	21	237	0.09	0.05	0.14	0.83	0.53	1.31	0.74	0.47	1.18
**Age**											
0 – 29	25	295	0.08	0.06	0.12	0.65	0.45	0.96	0.63	0.41	0.94
30 – 49	146	1118	0.13	0.11	0.15	1.00			1.00		
50 +	57	788	0.07	0.06	0.09	0.55	0.26	0.94	0.65	0.30	1.02
**Abdominal Surgery Risk Factors**											
Cholelithiasis	19	43	0.45	0.27	0.69				5.06	2.85	8.96
Inguinal Hernia	3	7	0.43	0.09	1.27				3.82	1.17	12.49
Diagnostic Procedures, Abdomen/Pelvis	3	5	0.61	0.13	1.79				4.34	1.32	14.22
Radiologic Small Bowel Exam	21	59	0.35	0.22	0.54				2.35	1.43	3.86
Urinary Tract Diagnostic Procedures	15	71	0.21	0.12	0.35				2.11	1.14	3.90
Gallbladder Radiography	19	50	0.38	0.23	0.60				2.82	1.69	4.69

^1^ Incidence rate (IR), events per person year.

^2^ Adjusted for age, gender, region, calendar year and quarter of cohort entry.

^3^ Adjusted for. age, gender, region, antidepressants, appendicitis, gallbladder surgeries, cholelithiasis, colonoscopy, irritable bowel syndrome, gastritis/duodenitis, hepatitis, disorders of liver, inguinal hernia, male genital surgery, MRI abdomen, diagnostic procedures, abdomen/pelvis, small bowel radiology, rectum/anus diagnostic procedures, urinary tract diagnostic procedures, urinary tract surgeries, gallbladder radiography, abdominal radiology, calendar year and quarter of cohort entry.

**Table 3:** Incidence of gallbladder surgery among tegaserod initiators and comparators (as-matched analysis)

**Variable**	**Events**	**Person years**	**I**.**R**.^**1**^	**95% Confidence interval**		**Hazard ratio**^**2**^	**95% Confidence interval**		**Hazard ratio**^**3**^	**95% Confidence interval**	
				**Low**	**High**		**Low**	**High**		**Low**	**High**
**Exposure**											
Tegaserod Initiators	36	1,115	0.03	0.02	0.04	0.98	0.62	1.55	0.99	0.62	1.58
Comparators	37	1,125	0.03	0.02	0.05	1.00			1.00		
**Gender**											
Female	67	1,999	0.03	0.03	0.04	1.00			1.00		
Male	6	242	0.02	0.01	0.05	0.76	0.33	1.75	0.50	0.20	1.24
**Age**											
0 – 29	9	299	0.03	0.01	0.06	0.77	0.56	1.21	0.77	0.56	1.22
30 – 49	45	1144	0.04	0.03	0.05	1.00			1.00		
50 +	19	797	0.02	0.01	0.04	0.61	0.55	0.81	0.61	0.52	0.83
**Gallbladder Surgery Risk Factors**											
Other Diseases of Appendix	1	2	0.49	0.01	2.71				13.60	1.77	401.41
Gallbladder or Bile Duct Surgeries	3	30	0.10	0.02	0.29				0.07	0.02	0.31
Acute Cholecystitis (575.0)	4	8	0.47	0.13	1.21				5.34	1.42	20.06
Cholelithiasis (574)	14	44	0.32	0.18	0.54				8.59	4.09	18.01
Constipation (564.0)	17	814	0.02	0.01	0.03				0.58	0.33	1.00
Abdominal Ultrasound	33	283	0.12	0.08	0.02				2.84	1.62	4.98
Radiographic Visualization, Gallbladder	13	51	0.25	0.13	0.43				3.31	1.63	6.71

^1^ Incidence rate (IR) per person year.

^2^ Adjusted for age, gender, region, and calendar year and quarter of cohort entry.

^3^ Adjusted for age, gender, region, and calendar year and quarter of cohort entry, number of abdominal pain physician visits, appendicitis, gallbladder surgery, cholecystitis, cholelithiasis, constipation, disorders of stomach and duodenum, liver surgery, peptic ulcer disease, sucralfate, abdominal ultrasound, and radiographic visualization of the gallbladder.

The effect of tegaserod on the incidence of abdominal or pelvic surgery was not different among the subset of the cohorts who were female (HR = 0.70, 95% C.I. = 0.53-0.92), or who had a baseline claim for constipation (HR = 0.62, 95% C.I. = 0.45-0.86), or who had a baseline claim for IBS (HR = 0.74, 95% C.I = 0.53-1.03) suggesting no effect measure modification by these characteristics. The lack of association between tegaserod and gallbladder surgery was also consistent across strata (HR = 1.02, 95% C.I. = 0.63-1.65 for women; HR = 0.98 95% C.I. = 0.58-1.65 for those with a baseline claim for constipation; HR = 1.04, 95% C.I. = 0.57-1.90 for those with a baseline claim for IBS). When we restricted the analysis of gallbladder surgeries to cholecystectomy, we found almost identical results (only 1 case of gallbladder surgery was not a cholecystectomy).

The “as-treated” analysis was also conducted separately for all abdominal surgeries (Table 4) combined and for gallbladder surgeries (Table 5). The incidence of all abdominal surgeries combined was similar in each of the different exposure categories in relation to tegaserod dispensing (current, recent, past, and non-use). The incidence rate of all abdominal surgeries, was not increased among either current tegaserod use (0.08 surgeries/person-year, RR = 0.68, 95% C.I. = 0.48-0.95) or past tegaserod use (0.07 surgeries/person-year, RR = 0.59, 95% C.I. 0.39-0.89) compared to non-use (0.12 surgeries/person-year, RR = 1.00, reference category). To the contrary, the incidence was statistically lower during these periods of tegaserod exposure compared to the non-use category, but not for the tegaserod recent-use exposure category (0.14 surgeries/person-year, RR = 1.17, 95% C.I. 0.76-1.80). For gallbladder surgery the rate of surgery per person year for the current-use group (0.03 surgeries/person-year, RR = 0.99, 95% C.I. = 0.56-1.77) and past-use group (0.02 surgeries/person-year, RR = 0.85, 95% C.I. = 0.42-1.72) was similar to the non-use group (0.03 surgeries/person-year, RR = 1.00, reference category) while the recent-use group was less similar, but remained statistically consistent (0.06 surgeries/person-year, RR = 1.78, 95% C.I. 0.88-3.59).

**Table 4:** Incidence rates and relative risks of abdominal or pelvic surgery (as-treated analysis)

**Variable**	**Events**	**Person years**	**I**.**R**.^**1**^	**95% Confidence interval**		**Rate ratio**^**1**^	**95% Confidence Interval**	
				**Low**	**High**		**Low**	**High**
**Tegaserod Use**								
Current	43	542	0.08	0.06	0.11	0.68	0.48	0.95
Recent	25	177	0.14	0.09	0.21	1.17	0.76	1.80
Past	28	393	0.07	0.05	0.10	0.59	0.39	0.89
Non-Use	132	1,090	0.12	0.10	0.14	1.00		
**Gender**								
Female	207	1,963	0.11	0.09	0.12	1.00		
Male	21	237	0.09	0.05	0.14	0.85	0.54	1.33
**Age**								
0 - 29	25	289	0.09	0.06	0.13	1.00		
30 - 49	144	1,110	0.13	0.11	0.15	1.47	0.96	2.26
50 +	59	802	0.07	0.06	0.09	0.85	0.53	1.36
**Surgery Risk Factors**								
Cholelithiasis (574)	19	43	0.45	0.27	0.69	3.25	2.00	5.28
Colonoscopy (45355–45387)	54	496	0.11	0.08	0.14	0.95	0.69	1.31
Irritable Bowel Syndrome (564.1)	89	910	0.10	0.08	0.12	0.88	0.67	1.15
Gastritis and Duodenitis (535)	47	312	0.15	0.11	0.20	1.37	0.98	1.91
Diagnostic Procedures^2^	80	437	0.18	0.15	0.23	1.92	1.45	2.55

^1^ Adjusted for gender, age, cholelithiasis, colonoscopy, irritable bowel syndrome, gastritis and duodenitis, and diagnostic procedures.

^2^ Composite variable consisting of MRI abdomen, other diagnostic procedures involving the abdomen or pelvis, radiologic small bowel examination, rectum or anus diagnostic procedures, urinary tract diagnostic procedures, radiographic visualization of the gallbladder, and abdominal radiologic examination.

**Table 5:** Incidence rates and relative risks of gallbladder surgery (as-treated analysis)

**Variable**	**Events**	**Person years**	**Incidence rate**^**1**^	**95% Confidence interval**		**Rate ratio**^**1**^	**95% Confidence interval**	
				**Low**	**High**		**Low**	**High**
**Tegaserod Use**								
Current	17	545	0.03	0.02	0.05	0.99	0.56	1.77
Recent	10	179	0.06	0.03	0.10	1.78	0.88	3.59
Past	10	401	0.02	0.01	0.05	0.85	0.42	1.72
Non-Use	36	1,115	0.03	0.02	0.04	1.00		
**Gender**								
Female	67	1,999	0.03	0.03	0.04	1.00		
Male	6	242	0.02	0.01	0.05	0.68	0.29	1.57
**Age**								
0 - 29	9	293	0.03	0.01	0.06	1.00		
30 - 49	44	1,135	0.04	0.03	0.05	1.35	0.66	2.77
50 +	20	813	0.02	0.02	0.04	0.87	0.39	1.91
**Gallbladder Surgery Risk Factors**								
Cholelithiasis (574)	14	44	0.32	0.18	0.54	8.51	4.23	17.14
Gallbladder or Bile Duct Surgeries	3	30	0.10	0.02	0.29	0.20	0.06	0.73
Constipation (564.0)	17	814	0.02	0.01	0.03	0.56	0.32	0.96
Diagnostic Procedures^2^	35	295	0.12	0.08	0.17	4.44	2.67	7.39

^1^ Adjusted for gender, age, cholelithiasis, gallbladder surgery, constipation, and diagnostic procedures.

^2^ Composite variable consisting of abdominal ultrasound and radiographic visualization of the gallbladder.

In these data, which reflect tegaserod use in actual clinical practice, we found no evidence for an increased risk of abdominal or pelvic surgery, or specifically gallbladder surgery, among persons who used the 5HT_4_ selective receptor partial agonist, tegaserod. This finding was consistently observed under different definitions of exposure to tegaserod and did not appear to be confounded by baseline imbalances between tegaserod initiators and their comparators. These results come from almost complete sampling of tegaserod initiators within a defined population with health insurance and represent usual patient care across a wide geographic area in the US; thus, we believe the results can be broadly generalized to tegaserod users. The explanation for the apparent elevation in risk of abdominal surgery during periods of recent tegaserod use is not apparent in the data available for this study. However, it may be related to clinical use of tegaserod for a short period if IBS is part of the initial differential diagnosis for a patient, and the drug is discontinued as the diagnostic picture evolves (perhaps with the introduction of evidence to support another diagnosis for which surgery is indicated) or a rebound of symptoms with discontinuation of tegaserod.

The Phase III clinical trial experience with tegaserod had suggested an incidence of abdominal surgeries of about one percent during approximately one-month of follow-up time, with gallbladder surgeries being a subset of that [1-4]. The higher absolute incidence of surgery that we observed in ZEST (4.1% for abdominal and pelvic surgeries and 1.3% for gallbladder surgeries) with a 6 month follow-up, likely reflects both the longer average follow-up time and differences between a clinical trial setting involving highly selected patients who meet study inclusion and exclusion criteria, and real-world clinical practice where tegaserod is prescribed to a broader mix of patients. The select subset of candidates for tegaserod therapy who qualify for clinical trials may be at a lower short-term risk of surgery than the patients who actually receive tegaserod in clinical practice. Even with the higher absolute incidence rate of surgeries observed in this study of tegaserod use in the “real-world”, we observed no increased risk for abdominal or pelvic surgery or for cholecystectomy among patients treated with tegaserod compared to matched non-tegaserod users.

Another study within this same insurance database found an incidence rate of 119/10,000 person-years for gallbladder surgery and 936/10,000 person-years for abdominal and pelvic surgery among persons with all forms of IBS [14]. Corresponding rates of 37/10,000 person-years for gallbladder surgery and 488/10,000 person-years for abdominal and pelvic surgery among similar persons selected from the health insurance population, but without a specific diagnosis of IBS, indicate that having IBS increases the risk of both of these surgery categories. An increased risk of abdominal and pelvic surgery, and specifically of cholecystectomy in patients with IBS, has been demonstrated by other research [15-18]. Notably, in IBS patients who undergo cholecystectomy, the presence of acute cholecystitis confirmed by tissue analysis of the resected gallbladder does not appear to be greater than that in patients without IBS [18]. These results suggest that gallbladder surgery may be used as a part of symptom evaluation in some patients. Certainly, there is no evidence that tegaserod affects gall bladder contraction or emptying [19].

Further, there may have been misclassification of IBS, particularly among the comparator cohort of this study, since the diagnosis codes for IBS have been found to be associated with substantial clinical heterogeneity [20,21]. If gallbladder surgery had been part of the symptom evaluation the IBS patients included in this study, there may have been distant past gallbladder surgery among some of the patients included, and these patients would be at lower risk of gallbladder surgery. Without access to a data source with extensive patient history to ascertain these remote gallbladder surgeries, the incidence estimates in this study may be underestimated as a result. If this underestimate of incidence were substantial, tegaserod effects on gallbladder surgery could be obscured (biased toward a null finding), but this effect would be considerably less pronounced for the outcome of general abdominal surgeries so the consistency of study results for these two outcomes is reassuring.

The incidence rates observed in the current study (323/10,000 person-years for gallbladder surgery and 854/10,000 person-years for abdominal and pelvic surgery among tegaserod initiators) suggest that those patients for whom tegaserod is prescribed are at elevated risk of later undergoing gallbladder surgery (although not abdominal or pelvic surgery) relative to those with IBS in earlier studies and at considerably increased risk of both categories of surgery when compared to the general population. The variation in reported incidence rates of abdominal surgery across studies underscores the importance of using an internal comparison group rather than an external or historical comparison group. Our matched comparison cohort was identified through propensity scores derived from health insurance claims so that it was not only contemporaneous with the tegaserod initiator cohort, but also was similar to it with respect to many baseline characteristics including demographics, diagnoses, procedures, drugs, and healthcare utilization. The similarity of the matched cohorts with respect to so many variables serves to address numerous potential confounding scenarios as alternate explanations for our findings. Any suggestion that the observed lack of increase in abdominal or pelvic surgery with tegaserod use is the result of a baseline difference between the compared groups would be predicated on the existence of a characteristic that predicts abdominal surgery and is sufficiently different between compared groups as to have obscured a real effect of tegaserod. Such a characteristic would have to be largely independent of the characteristics that were closely balanced between our study cohorts in order to have produced this effect, and the numerous variables that were part of the propensity score make this unlikely. Accordingly, the internal comparison of outcomes within this study should not be confounded by baseline differences among tegaserod users and comparators.

We identified study outcomes (abdominal and pelvic surgeries or gallbladder surgeries) during follow-up of the cohorts on the basis of claims for abdominal surgical services, a case screening process designed to have high sensitivity, since essentially all abdominal surgical procedures would be claimed through health insurance. We obtained medical records for most of the surgeries identified, and our surgery verification process had the effect of ensuring that all surgeries in this analysis met clinical criteria. This confirmation addresses the inherent limitation of insurance claims databases where the administrative purpose of a claim may result in a lack of correspondence between the claim and the underlying clinical condition. This case conformation also gives our study outcomes essentially complete specificity while retaining the high sensitivity of our original insurance claims screening.

The percentage of study outcomes that were confirmed upon review of the medical records reflects on the comprehensiveness of the initial screen where a wide range of surgical procedure claims brought to our attention many potential study outcomes including relatively minor surgeries and even non-surgical procedures that ultimately did not meet our study outcome definition when we reviewed the medical records. This comprehensive initial screen coupled with medical record review provides assurance of the completeness and accuracy of outcome ascertainment. The subset of potential study outcomes for which we could not obtain medical records was equally distributed among tegaserod and comparator cohorts and reflected an underlying mechanism that was largely administrative in nature (provider or facility refusal, archived or missing records, practice or facility closure). Accordingly, the estimated association between tegaserod use and study outcomes is not biased by differential outcome ascertainment, since the absolute incidence of study outcome is underestimated by a similar magnitude in the tegaserod and comparator cohorts.

## Conclusions

The results of the current study provide evidence that tegaserod does not increase the risk of either abdominal surgery or gallbladder surgery (including cholecystectomy), a finding that was consistently observed under different definitions of exposure, and did not appear to be confounded by any baseline differences of the tegaserod initiators.

## Appendix A: abdominal or pelvic surgery definition

A procedure performed under general endotracheal anesthesia by a surgeon (including a gynecologist or urologist) that involves entrance into the peritoneal cavity via incision with a scalpel, or via perforation with a laparoscopy trocar.Examples include:

a) Exploration of the peritoneal cavity

b) Partial or complete excision, repair, or transplantation of an abdominopelvic organ or body part

c) Repair of inguinal, femoral or incisional hernia

d) Placement or removal of a tube, catheter, or medical device

e) Lysis, vaporization, or cauterization of tissue

f) Evacuation of tissue, fluid, or foreign material

Or,A procedure performed under regional anesthesia (e.g., peripheral nerve, plexus, or neuraxial blockade) or general anesthesia, involving an incision into the vaginal or rectal wall of at least 1 cm in length, or penetration into the ischiorectal fossa.Examples include:

a) Hemorrhoidectomy (i.e., not rubber band ligation)

b) Repair procedures for pelvic organ prolapse, including stress urinary incontinence

c) Incision and drainage of perirectal abscess

This definition excludes endoscopic procedures by gastroenterologists, or other specialists, and procedures involving the insertion or removal of tubes/catheters, or aspiration of fluid/tissue, by radiologists, via percutaneous penetration of the peritoneal cavity with a large bore needle.Examples of Abdominal or Pelvic Surgical Procedures By OrganDistal esophagus

· Nissen fundoplication

· Total and distal esophagectomy

· Jejunal or colonic interposition for esophageal replacement

Stomach

· Partial or total gastrectomy

· Gastrostomy

· Gastrodudenostomy

· Pyloroplasty

· Vagotomy

· Gastric stapling or bypass for obesity

Small Intestine

· Ileostomy

· Jejunostomy tube placement

· Partial resections

· Intestinal bypass procedures

Appendix

· Appendectomy

Colon

· Colostomy

· Partial colectomy

· Total abdominoperineal resection

Rectum or Anus

· Total abdominoperineal resection

· Anterior resection of rectum

· Repair of rectal prolapse, involving an abdominal approach

Pancreas

· Pancreaticojejunostomy

· Partial or subtotal and total pancreatectomy

· Repair for trauma

· Cystogastrostomy and cystojejunostomy for pancreatic pseudocyst

Abdominal Wall (Hernia)

· Abdominal incisional hernias

· Inguinal hernias

Other Procedures Involving the Abdomen or Pelvis

· Splenectomy, partial or total

· Ventriculo-caval shunts

· Vascular bypass or repair procedures requiring a transabdominal approach (abdominal aorta, iliac arteries, etc.)

· Adrenalectomy

· Retroperitoneal, pelvic, and mesenteric lymph node dissections

Gallbladder or Bile Ducts

· Wedge biopsy

· Lobectomy or complete hepatectomy (i.e., for transplantation)

· Repair of liver injuries from trauma

· Drainage of abscesses, cysts, etc.

· Cholecystostomy

· Cholecystectomy

· Choledochojejunostomy

· Sphincteroplasty

· Portacaval shunts

Urinary Tract

· Partial and total nephrectomy

· Cystostomy

· Partial or total cystectomy

· Urinary diversion procedures

Female Genital

· Simple abdominal or vaginal hysterectomy with or without salpingo-oophorectomy

· Cesarean section

· Tubal sterilization procedure

· Cystocele repair with abdominal approach (e.g., Marshall Marchetti procedure, and not anterior or posterior colporrhaphy)

Male Genital

· Radical prostatectomy

· Radical orchiectomy

## Abbreviations

5HT_4_: Serotonin subtype 4; CI: Confidence interval; HR: Hazard ratio; IBS: Irritable bowel syndrome; IBS-C: Irritable bowel syndrome with constipation; ICD-9: International classification of diseases, 9^th^ revision; IR: Incidence rate; N: Number; RR: Relative risk; SAS: Statistical analysis system; ZEST: Zelnorm epidemiological study.

## Competing interests

This study was conducted by OptumInsight Epidemiology under a research contract with Novartis Pharmaceuticals Corporation. The contract granted OptumInsight oversight of the study conduct, reporting, and interpretation, as well as final wording of any resulting manuscripts. At the time of the study, David Earnest and Elena Rivero were employees of Novartis, and John Seeger, Sherry Quinn, and Alec Walker were employees of OptumInsight Epidemiology.

## Authors’ contributions

JS developed the study protocol, ensured adherence of the study to the protocol, and drafted the manuscript. SQ carried out the statistical analyses, participated in protocol development and manuscript review. DLE participated in protocol development and manuscript review. AL provided input to the protocol, clinical review of study outcomes, and manuscript review. BK provided input to the protocol, clinical review of study outcomes, and manuscript review. ER provided input to the protocol and manuscript review AMW conceived of the study, and participated protocol development and manuscript review. All authors read and approved the final manuscript.

## Pre-publication history

The pre-publication history for this paper can be accessed here:

http://www.biomedcentral.com/1471-230X/12/171/prepub
